# A comparison of the different 3D CT scanning modes on the GTV delineation for the solitary pulmonary lesion

**DOI:** 10.1186/1748-717X-9-211

**Published:** 2014-11-12

**Authors:** Dong-ping Shang, Cheng-xin Liu, Yong Yin

**Affiliations:** Department of Radiation Oncology, Shandong Tumor Hospital, Shandong Province, 250117 China; Department of Radiation Oncology, Shandong Tumor Hospital, 440 Jiyan Road, Jinan, 250117 China

**Keywords:** CT scan, Solitary pulmonary lesion, Gross tumor volume

## Abstract

**Objectives:**

To investigate the impacts of the different three-dimensional CT (3DCT) scanning modes on the GTV delineation for solitary pulmonary lesion (SPL) based on four-dimensional CT (4DCT), and to evaluate the feasibility of using the spiral CT scan in CT simulation.

**Materials and methods:**

Twenty-one patients with SPL underwent axial CT scan, spiral CT scan and 4DCT simulation scan during free-breathing, respectively. The same clinical radiation oncologist delineated the gross tumor volume (GTV) under the same CT window setting. GTV_A_ and GTV_S_ were created from the axial and spiral images, respectively. ITV_MIP_ was created from the maximum intensity projection (MIP) reconstructed 4D images. The target volumes and position between GTV_A_, GTV_S_ and ITV_MIP_ were compared. The matching index (MI) between GTV_A_ and GTV_S_, and the correlation between MI and GTV_S_ were evaluated.

**Results:**

ITV_MIP_ was significantly larger than GTV_A_ and GTV_S_ (*p*_*s*_ = 0.000). The ratios of ITV_MIP_ to GTV_A_ and GTV_S_ were 1.57 ± 0.54 and 1.66 ± 0.61, respectively. There was no significant difference between GTV_A_ and GTV_S_(*p* = 0.16). A comparison of the centroidal positions in x, y, and z directions for GTV_A_, GTV_S_ and GTV_4Dmip_ showed no significant difference (*p*_x_ = 0.17, *p*_y_ = 0.40, *p*_*z*_ = 0.48). Additionally, there was no difference between distances from the centroidal positions of GTV_A_ and GTV_S_ to the origin of coordinates (*p* = 0.51). MI between GTV_A_ and GTV_S_ was 0.41 ± 0.24 (range 0–0.89), and it was positively correlated with the tumor volume (*r* = 0.64, *p* = 0.002).

**Conclusion:**

There was no impact on the volume or centroidal position of GTV by the axial scan or spiral scan in 3DCT simulation for SPL. MI between GTV_A_ and GTV_S_ was small. A positively correlation was found between MI and GTV_S_. Relative to axial scanning mode, spiral CT scan was more timesaving and more efficient, it was feasible in 3DCT simulation for SPL.

## Introduction

The aim of radiation treatment is to increase the radiotherapy effects on the tumor while avoiding excessive toxicity [1, 2]. Computed tomography (CT) simulation and accurately determining the target margin were prerequisite for the successful oncology radiotherapy. Three-dimensional CT (3DCT) and four-dimensional CT (4DCT) simulation techniques are both in used, and 4DCT simulation is currently the leading simulation technology. It has enabled CT data acquisition to be correlated to the respiratory cycle(s), and allows a series of 3DCT data sets to be acquired at a number of points in a patient’s breathing cycle offering visualization of tumor motion on a patient by basis [3–5]. However, the 3DCT simulation is still widely used for 3D conformal radiotherapy (3D-CRT) at present. There were two scanning modes in 3DCT simulation, which were axial and spiral scanning mode. Because the speed of tube rotation in axial mode was slower than that in spiral mode, we often treat head and neck tumors that are not affected by respiratory motion with spiral scan [6]. For thoracic and abdominal tumor, respiratory motion and heartbeat are important factors to determine the gross tumor volume (GTV). The slow speed scanning mode could collect more respiratory motion information, so we usually adopted axial scanning mode for simulation during free-breathing [7]. But whether the axial scan and spiral scan that are usually adopted would influence the GTV of the lung tumor in motion during free-breathing was unclear. Basing on 4DCT technique, this study evaluates how the axial and spiral scanning modes affect the position, volume and spatial match relationships on the GTV delineation for the solitary pulmonary lesion (SPL) in 3DCT simulation.

## Materials and methods

### Patients

From March 2010 to September 2012, 21 patients with pathologically proven peripheral lung cancer or pulmonary metastasis who were treated with stereotactic body radiotherapy (SBRT) at Shandong Tumor Hospital were included in this study. Institutional Review Board of the hospital approval and informed consent were obtained for the present study. All patients had SPL with no adhesion between the tumor edge and pleura. And all of them could breathe freely and cooperate with CT scanning. The subjects consisted of 13 males and 8 females. The age of the patients ranged from 38 to 78 years, with a median age of 59 years. The lesion volumes ranged from 1.45 cm^3^ to 35.41 cm^3^, located in left lung in 9 cases and right lung in 12 cases, respectively.

### CT simulation and image acquisition

The patients were immobilized in the supine position with their arms raised above head using vacuum bags. First, the axial scan was performed during free-breathing using Philip Brilliance big bore CT scanner (Philips Medical Systems, Inc., Cleveland, OH, USA). And then patients were conducted spiral scan and 4D scan consecutively, the image layer thickness was set to 3 mm. The axial scan cycle (scan + in-couch time) was 2.8 s. The pitch was 0.938 under spiral scanning. 4D technique was a kind of slow spiral scan mode, which would adjust the pitch according to respiratory rate in 4D scanning (pitch: 0.09 ~ 0.15) [8]. The respiratory signal was recorded with the Real-Time Position Management (RPM, Varian Medical Systems, Palo Alto, CA, USA) by tracking the trajectory of the infrared markers placed on the patient’s abdomen [9]. The respiratory signal was transferred to the CT scanning device, where the respiratory phase was matched with the acquired image(s). The 4D volume data were reconstructed into ten sequence images of different respiration phase (0%, 10%, 20% …90%) according to the respiration signal acquired by the RPM system, the 0% phase corresponded to the end-inhalation and the 50% phase corresponded to the end-exhalation. The ten sequence images were reconstructed with the same thickness of 3 mm.

### Delineation, comparison and matching of GTV

**Figure 1:**
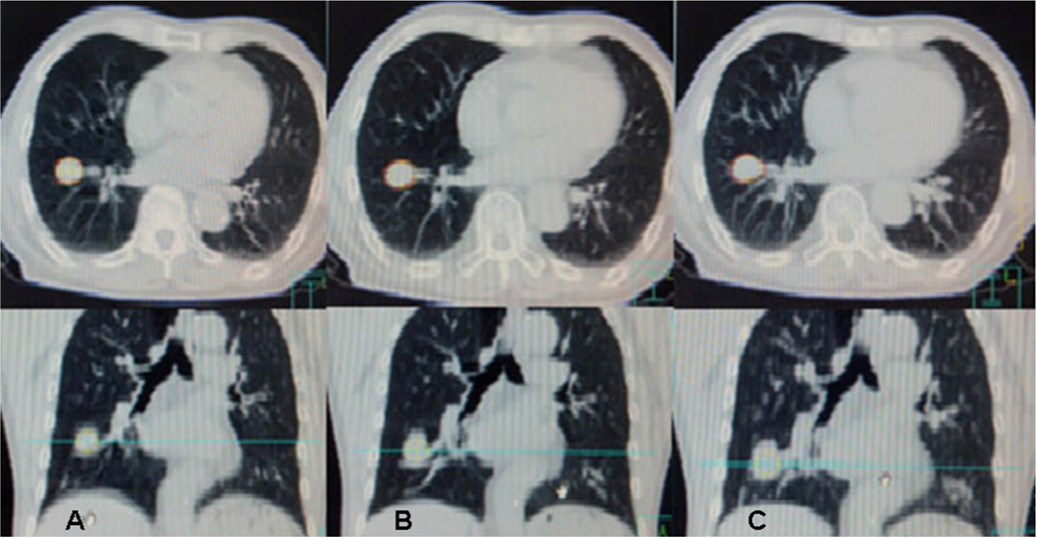
**GTV**_**A**_ **, GTV**_**S**_ **and ITV**_**MIP**_ **were created from the three sets images. A**: axial scan, **B**: spiral scan, **C**: 4D scan, respectively. And the coronal sections were reconstructed at the same time.

The images obtained by three scanning modes were processed by Tumor Loc Software under the same CT window setting (W: 1600 C:-600). GTV_A_ and GTV_S_ were created from the two sets of CT images obtained by axial and spiral scanning modes, respectively (Figure 1A and B). The 3D coordinates of GTV_A_ were Ax, Ay and Az. The coordinates of GTV_S_ were Sx, Sy and Sz. calculating the distance from the centroidal position of GTV_A_ and GTV_S_ to the origin of coordinates, respectively. Tumor Loc software was used to retrospectively create the maximum intensity projection (MIP), and the ITV_MIP_ was created from the MIP images [10] (Figure 1C). Using the Eclipse 8.6 treatment planning system, by which would conduct target registration on GTV_A_ and GTV_S_, then calculate the matching index (MI) between GTV_A_ and GTV_S_.MI = (GTV_A_∩GTV_S_)/(GTV_A_∪GTV_S_) (∩and ∪ were intersection and union, respectively) [11].

### Statistical analysis

All statistical analyses were performed using the SPSS17.0 package (SPSS Inc, Chicago, IL, USA). The Wilcoxon test was used to compare the tumor volumes and the isocenter coordinates (x, y, z axial) gained by different scanning modes. A Pearson correlation analysis was utilized to study the relationship between MI and GTV_S_. Differences were considered to be significant if the *p*-value was less than 0.05.

## Results

The average ± standard deviation of GTV_A_ and GTV_S_ volumes were 9.94 ± 9.74 cm^3^ (range 1.72 ~ 36.56 cm^3^) and 10.14 ± 10.25 cm^3^ (range 1.45 ~ 35.41 cm^3^), respectively. There was no statistically significant difference between the volume of GTV_A_ and GTV_S_ (*p* = 0.16) (Table 1). The volume of ITV_MIP_ was 14.33 ± 12.47 cm^3^ (rang 2.93 ~ 41.16 cm^3^). On pair wise analysis using Wilcoxon rank test, ITV_MIP_ was significantly larger than GTV_A_ and GTV_S_ (*p*_*s*_ = 0.000) (Table 1), and the ratios of ITV_MIP_ to GTV_A_ and GTV_S_ were 1.57 ± 0.54 and 1.66 ± 0.61, respectively.

**Table 1:** **Statistical comparison of tumor volume according different scanning modes**

Comparison	***p***-value
GTV_A_ *vs* GTV_S_	0.159
ITV_MIP_ *vs* GTV_A_	0.000
ITV_MIP_ *vs* GTV_S_	0.000

The center of tumor coordinates for GTV_A_, GTV_S_ and ITV_MIP_ were listed in Table 2 and the averages ± standard deviations were showed in Table 3. No differences were determined in x, y and z directions (*p*_x_ = 0.17, *p*_y_ = 0.40, *p*_*z*_ = 0.48). The distances from the center of GTV_A_ and GTV_S_ to the origin of coordinate were 36.94 ± 24.64 cm and 36.88 ± 24.39 cm, respectively. There are no difference between the two distances (*p* = 0.51).

**Table 2:** **The center of tumor coordinates for GTV**_**A**_ **, GTV**_**S**_ **and ITV**_**MIP**_

case	GTV _A_			GTV _S_			ITV _MIP_		
	x	y	z	x	y	z	x	y	z
1	-7.69	-71.84	-8.5	-7.56	-70.14	-8.33	-7.62	-70.89	-8.67
2	-7.46	-70.79	-4.92	-7.67	-69.39	-5.02	-7.68	-69.84	-4.81
3	7.89	-76.79	-8.03	7.89	-77.34	-7.78	7.85	-77.34	-8.03
4	-4.49	-57.69	-0.74	-4.44	-57.89	-0.56	-4.45	-57.84	-0.73
5	-7.58	-57.59	0.72	-7.41	-57.79	0.72	-7.49	-57.69	0.86
6	-2.8	-49.44	-5.92	-2.75	-49.44	-5.75	-3.02	-49.34	-5.88
7	-7.21	-66.69	-10.81	-7.07	-65.89	-10.91	-7.17	-66.04	-10.61
8	3.64	7.51	-0.52	3.49	7.86	-0.33	3.69	7.66	-0.67
9	-9.93	-33.24	2.17	-10.14	-33.39	2.12	-10.26	-33.24	2.34
10	8.52	-24.84	-5.35	8.88	-25.29	-5.18	8.5	-24.64	-5.33
11	-3.41	-18.39	5.74	-3.33	-18.69	5.96	-3.59	-18.64	5.66
12	-9.57	-10.49	-9.92	-9.57	-10.74	-9.71	-9.59	-10.79	-9.93
13	5.8	-4.29	-3.36	6.07	-4.39	-3.52	5.71	-4.3	-3.44
14	10.47	-8.69	0.59	10.4	-8.24	0.97	10.48	-8.69	0.74
15	-4.01	-16.59	-7.27	-3.94	-16.89	-7.08	-4.15	-15.39	-7.37
16	-8.35	-3.59	-5.29	-8.38	-4.09	-5.43	-8.08	-4.04	-5.4
17	9.75	-50.69	-5.45	9.86	-51.09	-5.01	9.93	-51.59	-5.02
18	-5.83	-59.24	-3.81	-5.94	-59.19	-3.71	-5.81	-59.34	-4.00
19	5.62	18.86	-3.1	5.28	18.36	-3.67	5.55	18.51	-3.32
20	-5.41	-9.56	-1.86	-5.31	-9.41	-1.95	-5.49	-9.96	-2.07
21	-4.49	-18.39	-7.18	-4.57	-18.64	-7.31	-4.62	-18.34	-7.16

**Table 3:** **The comparison of 3D coordinates for GTV**_**A**_ **, GTV**_**S**_ **and ITV**_**MIP**_ **(**  **±s)**

Scan modes	x axial	y axial	z axial
GTV_A_	-1.74 ± 6.99	-32.50 ± 29.07	-3.94 ± 4.20
GTV_S_	-1.72 ± 7.02	-32.46 ± 28.83	-3.88 ± 4.22
ITV_MIP_	-1.78 ± 7.02	-32.47 ± 28.95	-3.94 ± 4.20
*x* ^*2*^	3.51	1.83	1.49
*p*	0.17	0.40	0.48

**Figure 2:**
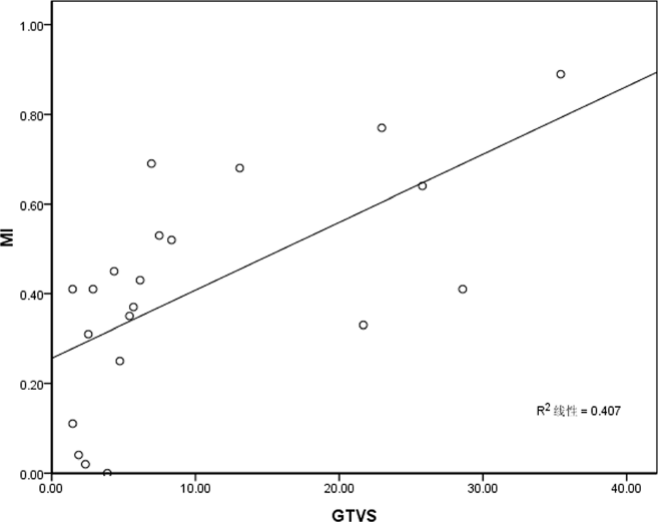
**Correlation between MI and GTV**_**S**_ **.**

MI between GTV_A_ and GTV_S_ was 0.41 ± 0.24; the variation range was from 0 to 0.89. The correlation between the MI and GTV_S_ was illustrated in Figure 2. There was a positive correlation between MI and GTV_S_. The correlation coefficients was (*r* = 0.64 *p* = 0.002).

## Discussion

SBRT is a safe and effective alternative therapy for patients with the inoperable lung cancer or metastatic tumor [12]. Physiologic motions such as respiratory motion and heart beat would lead to tumor displacement and change in shape, so delineation of the GTV was a critical component in the radiotherapy process for thoracic tumor [2]. Investigators have determined the target margin of thoracic tumor using the technology of active breathing control and 4DCT, which could reduce the interference on radiotherapy and increase the irradiation dose in target margin [13–16].

4DCT allowed for the clear delineation of the anatomical structures during free- breathing. This technique is widely used in evaluating the displacement of tumor and organ for thoracic and abdominal cancers. The ten sequence images reconstructed by 4D volume data could demonstrate movements of the tumor dynamically, and the ITV_MIP_ represented the volume where tumor was present at any time within the respiratory cycle, which contained motion information of the tumor [9, 17–19]. The axial scanning mode was scanning alternates with the movement of scanner couch, that was, “scanning-in couch-scanning” pattern. As a result, the simulation time was long. Taking the ordinary 16 layers CT for example, the X -ray tube needs 1 s to rotate 360 degree and the scan cycle was 2.8 s by the axial mode, the scanning range was 24 mm (with 16 × 1.5 mm detector). The spiral mode was continuous scanning with CT scanner couch moving at a speed of 30 mm/s. There was no interval during the scanning process. It takes only 0.75 s to complete the scan rang of 24 mm in superior-inferior direction. In this study, we recorded and compared the scanning time for the same range by axial and spiral mode, respectively. The result revealed that axial mode took a longer time to complete the same scan than spiral mode, T_A_ = 28.93 ± 4.63 s, T_S_ = 8.81 ± 1.41 s (*p* = 0.000). Furthermore, the spiral mode could reduce motion artifacts, and increase the accuracy of tumor shape and position. Though the high speed of spiral mode, the scanning time for region of interest (ROI) was short for the small lesions and GTV contains little amount of respiratory motion information. The respiratory rate of the patients enrolled in the study was 16.00 ± 2.81breaths/min, and the respiratory cycle was 3.87 ± 0.73 s. The axial and spiral scans often complete ROI within 1 s. The key point was the ratio of the scanning time for ROI to the respiratory cycle. The higher the ratio was, the more motion information was included in the images. Among our subjects, there were 14 patients whose GTV_A_ was larger than GTV_S_, while 7 smaller. There was no statistically significant difference in volume between the two scanning modes. We analyzed the tumor motion information included in GTV_A_ and GTV_S_ by comparing the volumes of ITV_MIP_ with GTV_A_ and GTV_S_, respectively. The ratio between ITV_MIP_ and GTV_A_ was 1.57 ± 0.54 (*p* = 0.000). It suggested that the tumor motion information included in GTV_A_ was limited. The result was similar to the reports of Nakamura M [20, 21]. There was statistically significant difference in volume between ITV_MIP_ and GTV_S_ (*p* = 0.000). The ratio between ITV_MIP_ and GTV_S_ was 1.66 ± 0.61, which suggested that the motion information included in GTV_S_ by spiral mode was limited too. The result of this study revealed that there was no difference in the volume of GTV by axial or spiral mode in 3DCT simulation. The tumor was moving when the axial and spiral scanning was performed during free-breathing. The GTV_3D_ (GTV_A_ and GTV_S_) created from the axial and spiral images included little respiration-induced motion information. It only represented the instant shape and volume that comes randomly by axial or spiral mode during the respiratory cycle.

ITV_MIP_ was significantly larger than GTV_A_ and GTV_S_. The differences in volume between GTV_3D_ and ITV_MIP_ were caused by the tumor motion in three dimensions. The motion information was included in ITV_MIP_. The motion amplitude in superior-inferior direction was larger than those in lateral and anterior-posterior directions. The superior-inferior motion of GTV centroid in lower lobe was larger than the in upper lobe [22, 23]. So the internal target volume (ITV) was generated from GTV_3D_ by adding different margins in x, y and z directions. The compensated volume was still larger than ITV_MIP_ created by 4D images.

The axial and spiral scan were all performed during free-breathing. The centroid coordinates of GTV_A_ and GTV_S_ were instant location during the respiratory cycle. There was no statistically significant difference in 3D coordinates of GTV_A_ and GTV_S_, nor the distances from the centroidal positions to origin of coordinates. What’s more, there was no difference in the centroidal position between GTV_3D_ and ITV_MIP_ (*p*_x_ = 0.17, *p*_y_ = 0.40, *p*_*z*_ = 0.48). This suggests that there was no obvious impact on for the tumors’ central position by the three scanning modes during free-breathing.

Although there was no difference in volume and the centroidal coordinates between the axial and spiral modes during free-breathing, there was still respiration-induced change in shape of tumor [24, 25]. We conducted target registration on GTV_A_ and GTV_S_, MI range from 0 to 0.89. If the two target regions coincide completely, MI was 1, otherwise, that was 0. There was one case with no coincidence in our data, which suggested an obvious tumor displacement, and/or the tumor shapes varied greatly. Furthermore, there was a linear correlation between MI and GTV_S_ (*r* = 0.64, *p* = 0.002). The small volume of GTV was one of the reasons for MI declination. The variation of MI was large in individuals. The low MI didn’t mean increase the margins, and different MI didn’t mean asymmetric margin either, because MI wasn’t determined by the displacement and the change in shape, it was affected by the tumor volume also.

## Conclusion

In summary, our study found no impact on the volume and centroidal position of GTV by the axial or spiral scan in 3DCT simulation. There were no difference in the centroidal positions between 3DCT and 4DCT as well. The tumor motion information included in GTV_A_ and GTV_S_ was both limited. A linear correlation was found between MI and GTV_S_. Relative to axial scanning mode, spiral scan was more timesaving, reduced some motion artifacts and increased the accuracy of GTV due to its high scanning speed. So the spiral mode was a reasonable option to replace axial scan in 3DCT simulation for SPL.

## Abbreviations

CT: Computed tomography
3DCT: Three-dimensional CT
SPL: Solitary pulmonary lesion
4DCT: Four-dimensional CT
3D-CRT: Three-dimensional conformal radiotherapy
GTV: Gross tumor volume
GTV_A_: Gross tumor volume gained by axial scan
GTV_S_: Gross tumor volume gained by spiral scan
ITV_MIP_: Internal target volume gained by 4D maximum intensity projection
MIP: Maximum intensity projection
SBRT: Stereotactic body radiotherapy
RPM: Real-Time Position Management.
MI: Matching index
T_A_: Time of completing the scan by axial mode
T_S_: Time of completing the scan by spiral mode
ROI: Region of interest
GTV_3D_: GTV_A_ and GTV_S_
ITV: Internal target volume.
